# Crossing the Wall: Characterization of the Multiheme Cytochromes Involved in the Extracellular Electron Transfer Pathway of *Thermincola ferriacetica*

**DOI:** 10.3390/microorganisms9020293

**Published:** 2021-01-31

**Authors:** Marisa M. Faustino, Bruno M. Fonseca, Nazua L. Costa, Diana Lousa, Ricardo O. Louro, Catarina M. Paquete

**Affiliations:** Instituto de Tecnologia Química e Biológica António Xavier, Universidade Nova de Lisboa, Av. da República, 2780-157 Oeiras, Portugal; marisa.mfaustino@gmail.com (M.M.F.); bfonseca@itqb.unl.pt (B.M.F.); nazuacosta@itqb.unl.pt (N.L.C.); dlousa@itqb.unl.pt (D.L.); louro@itqb.unl.pt (R.O.L.)

**Keywords:** *Thermincola*, multiheme c-type cytochromes, extracellular electron transfer, electroactive organisms, Gram-positive bacteria

## Abstract

Bioelectrochemical systems (BES) are emerging as a suite of versatile sustainable technologies to produce electricity and added-value compounds from renewable and carbon-neutral sources using electroactive organisms. The incomplete knowledge on the molecular processes that allow electroactive organisms to exchange electrons with electrodes has prevented their real-world implementation. In this manuscript we investigate the extracellular electron transfer processes performed by the thermophilic Gram-positive bacteria belonging to the *Thermincola* genus, which were found to produce higher levels of current and tolerate higher temperatures in BES than mesophilic Gram-negative bacteria. In our study, three multiheme *c*-type cytochromes, Tfer_0070, Tfer_0075, and Tfer_1887, proposed to be involved in the extracellular electron transfer pathway of *T. ferriacetica*, were cloned and over-expressed in *E. coli*. Tfer_0070 (ImdcA) and Tfer_1887 (PdcA) were purified and biochemically characterized. The electrochemical characterization of these proteins supports a pathway of extracellular electron transfer via these two proteins. By contrast, Tfer_0075 (CwcA) could not be stabilized in solution, in agreement with its proposed insertion in the peptidoglycan wall. However, based on the homology with the outer-membrane cytochrome OmcS, a structural model for CwcA was developed, providing a molecular perspective into the mechanisms of electron transfer across the peptidoglycan layer in *Thermincola*.

## 1. Introduction

Extracellular electron transfer (EET) is a metabolic process that allows microorganisms to exchange electrons with external conductive solids, including metal oxides in their natural environment and electrodes in bioelectrochemical systems (BES) [1,2]. Microorganisms capable of producing electricity were reported more than one century ago [3]. The ability of these microorganisms to exchange electrons with electrodes earned the designation of electroactive but only recently their EET mechanisms have been explored in detail [1,4,5,6,7]. There is a great interest in understanding the EET mechanisms of electroactive organisms due to their capacity to produce electricity and added-value products from renewable and carbon-neutral sources (e.g., municipal wastewater), under ambient temperature and pressure and with low greenhouse gas emissions contributing to tackle numerous societal challenges including clean energy and global warming [8,9,10,11,12].

Currently, more than 100 organisms are known to be electroactive [2,13]. Among these, the thermophilic iron-reducing bacterium *Thermincola potens* strain JR, identified in a microbial fuel cell (MFC) operating at high temperatures (55 °C), was shown to produce higher levels of current than the well-studied mesophilic Gram-negative electroactive bacteria *Geobacter sulfurreducens* and *Shewanella oneidensis* MR-1 (0.42 mA versus 0.25 mA and 0.03–0.30 mA for *Geobacter* and *Shewanella* respectively, in the same type of MFC) [14]. Since BES operating at high temperatures enable the production of higher current densities and allow higher chemical oxygen demand (COD) removal [14,15,16], the understanding of the EET processes performed by these thermophilic electroactive organisms has gained impetus in recent years [16,17,18]. 

*T. potens* JR shares 99% sequence similarity with *T. ferriacetica* [19], that was the first Gram-positive bacterium found to perform direct electron transfer to an electrode in BES [20,21]. Since *T. ferriacetica* grows at faster rates and to higher optical densities than *T. potens* [19,22], this strain has been considered a more promising organism for investigation within its genus. Indeed, *T. ferriacetica* produces thicker biofilms (>150 μm) and has a higher surface area for connecting with the electrode, which results in higher current densities when compared with *T. potens* [23,24]. Furthermore, the presence of a conductive extracellular matrix in *T. ferriacetica* [17,23,25] and the absence of one in *T. potens* suggests that this species may have redox active proteins in the extracellular matrix as observed for *Geobacter* [26,27].

Electroactive organisms contain specific pathways to establish electrical contact between intracellular carriers and the cell surface [7,28,29]. The current state of the art shows multiheme *c*-type cytochromes (MHC) as key players in these pathways, transferring electrons across the cell envelope of numerous bacteria [28,29,30]. EET can occur through direct contact between the MHC and the external electron donor or acceptor [18,31,32,33,34], or indirectly, where soluble electron shuttles (e.g., flavins, quinones) mediate electron transfer between the cell and the terminal electron acceptor [35,36,37,38,39]. 

In *T. potens*, an EET pathway composed by four MHC was proposed to transfer electrons to extracellular acceptors [34]. These proteins are conserved between the two *Thermincola* species: TherJR_1117 (Tfer_0070 in *T. ferriacetica*) that contains 10 heme binding motifs (CXXCH, where X is any amino acid) and is predicted to be anchored to the membrane; TherJR_0333 (Tfer_1887) that also contains 10 heme binding sites and is located in the periplasm; TherJR_1122 (Tfer_0075) that contains six heme binding motifs and is proposed to be embedded in the peptidoglycan cell-wall; and TherJR_2595 (Tfer_3193) that has 9 heme binding sites and is located at the cell surface. The structure and functional mechanisms of the cell-surface MHC OcwA from *T. potens* (TherJR_2595) were elucidated, demonstrating that this protein can transfer electrons to an electrode and reduce soluble electron shuttles and oxyanions [18]. Furthermore, the structure of OcwA showed that the overall fold and organization of the hemes is more similar to MHC involved in the biogeochemical cycles of nitrogen and sulfur [18], instead of cell-surface MHC of *Shewanella* and *Geobacter* genera involved in EET [33,40].

To characterize the proposed EET pathway of *Thermicola sp.*, we have cloned and heterologously expressed the MHC from *T. ferriacetica* in *Escherichia coli*, hereafter referred to as: Inner-membrane decaheme cytochrome A (ImdcA, Tfer_0070), Periplasmic decaheme cytochrome A (PdcA, Tfer_1887) and Cell-wall cytochrome A (CwcA, Tfer_0075). The purification of ImdcA and PdcA along with their electrochemical and biochemical characterization, supports a pathway of EET via these two proteins. Sequence homology modelling was used to propose the three-dimensional architecture of CwcA, showing a novel potential pathway for electrons to be transferred across the thick peptidoglycan cell-wall of *Thermincola* sp.

## 2. Materials and Methods

### 2.1. Bacterial Strains and Growth Conditions 

#### 2.1.1. ImdcA

The gene *imdcA* (Tferr_0070, NZ_LGTE01000001.1) was synthesized (NZYTech, Lisbon, Portugal) and amplified using the primers Forw_Tfer0070 and Rev_Tfer0070 presented in Table S1. Cloning into the expression vector pBAD/Thio-TOPO (Invitrogen, Carlsbad, CA, USA) was achieved using the methodology described by the manufacturer. To facilitate protein purification, the C-terminal polyhistidine (6× His) tag present in the plasmid was replaced by a Strep-tag^®^ (WSHPQFEK aminoacid sequence) through site-directed mutagenesis using the primers strep_forw and strep_rev listed in Table S1. The resulting plasmid was transformed in *E. coli* JM109(DE3) strain, previously transformed with the vector pEC86 that encodes the cytochrome *c* maturation system (Ccm). These cells were then grown aerobically at 37 °C and 150 rpm in an Erlenmeyer flask with 2/5 of its capacity filled with Terrific Broth (TB) medium supplemented with 34 µg mL^−1^ chloramphenicol and 100 µg mL^−1^ ampicillin. A 1% inoculum of cells grown overnight under the same conditions was used to initiate growth. Protein expression was induced by the addition of 1 mM of L-arabinose at mid-exponential phase (after approximately 6 h of growth), and the cells were allowed to grow for another 16 h at 30 °C. The cells were then harvested by centrifugation at 11,300× *g* for 8 min at 4 °C.

#### 2.1.2. PdcA

The plasmid pBAD202/D-TOPO harboring *pdcA* gene (Tfer_1887, LGTE01000012.1) was obtained through site-directed mutagenesis, using pBAD202/D-TOPO harboring TherJR_0333 [41] and the primers listed in Table S1 (Tfer1887_mutX_forw and Tfer1887_mutX_rev, where “X” represents the number of the mutation). The resulting plasmid was transformed in *E. coli* BL21(DE3) previously transformed with vector pEC86. PdcA was expressed as described for ImdcA, however the TB medium was supplemented with 50 µg mL^−1^ kanamycin instead of ampicillin.

#### 2.1.3. CwcA

The gene *cwcA* (Tferr_0075, NZ_LGTE01000001.1) was amplified and cloned in pBAD/Thio-TOPO (Invitrogen) as described by the manufacturer and in [41] using the primers listed in Table S1 (Forw_STC_0075, Forw_STC_pBADthio, Rev_Tfer0075 and Rev_STC_0075). In this construct, the native signal peptide of CwcA was replaced by the signal peptide of the small tetraheme cytochrome (STC) from the Gram-negative *S. oneidensis* MR-1. The resulting plasmid was transformed in *E. coli* BL21(DE3) previously transformed with vector pEC86. CwcA was expressed as described for ImdcA. All constructs were confirmed by DNA sequencing (Eurofins, Ebersburg, Germany).

### 2.2. Protein Purification

#### 2.2.1. ImdcA

To purify ImdcA the cell pellet was first resuspended in 20 mM Tris-HCl buffer (pH 9.0), containing EDTA-free Protease Inhibitor Cocktail (Merck, Mannheim, Germany) and DNase I (Merck), using a 1:1 ratio of cell wet weight to buffer volume. Cells were then lysed using a French press at a pressure of 1000 psi. To solubilize the proteins from the membranes, the membrane fraction, collected by centrifugation at 200,000× *g* for 1 h at 4 °C, was resuspended in 20 mM Tris-HCl buffer (pH 9.0) containing 100 mM NaCl and 2% SB-12, and left at 4 °C overnight with gentle stirring. This mixture was then centrifuged at 200,000× *g* for 1 h at 4 °C, and the supernatant containing solubilized proteins was loaded in a Strep-Tactin^®^ Superflow^®^ high-capacity cartridge (IBA) previously equilibrated with 100 mM Tris-HCl buffer (pH 9.0) containing 150 mM NaCl and 0.2% SB-12. The loaded sample was washed with the same buffer and eluted in buffer containing 5 mM D-desthiobiotin (IBA, Göttingen, Germany). After elution, the proteins were dialyzed overnight in 20 mM Tris-HCl buffer (pH 9.0) with 100 mM NaCl and 0.2% SB-12 or 0.5% Triton X-100, and analyzed for protein purity by SDS-PAGE 12% and UV-visible spectroscopy.

#### 2.2.2. PdcA

To purify PdcA the cells were resuspended in 200 mM Tris-HCl buffer (pH 7.6) containing 0.5 M sucrose, 0.5 mM EDTA and 100 mg.L^−1^ lysozyme and supplemented with EDTA-free protease inhibitor cocktail (Merck) and DNase I (Merck). This mixture was stirred for 1 h at 4 °C. The periplasmic fraction was separated by centrifugation at 11,300× *g* for 8 min at 4 °C, and dialyzed in 50 mM Tris-HCl buffer (pH 9.0) overnight at 4 °C. The protein was precipitate at room temperature using a gradient (20% to 50%) of an ammonium sulfate concentrated solution (3.5 M) (Sigma, Steinheim, Germany). After each salt addition the precipitate was collected by centrifugation at 5000× *g* for 5 min. Each pellet was solubilized in 20 mM Tris HCl buffer (pH 9.0) containing 100 mM KCl and 10 mM sodium cholate, and analyzed by SDS-PAGE 12% to select the fraction that contained the desired protein. The fraction containing PdcA was precipitated at approximately 30% of ammonium sulfate. This fraction was then dialyzed overnight in 20 mM Tris HCl buffer (pH 9.0) with 10 mM sodium cholate, to prevent precipitation of the target protein [41], and loaded onto a Q-sepharose HP column (GE Healthcare, Uppsala, Sweden) previously equilibrated with the same buffer and a salt gradient from 0 to 1 M was applied. The PdcA protein was eluted in the flow through. The fractions were analyzed for protein purity by SDS-PAGE 12% and UV-visible spectroscopy.

### 2.3. Mass Spectrometry

The protein solution was desalted and concentrated using POROS C8 (3 M Empore, St. Paul, MN, USA) and eluted directly onto the MALDI plate using 0.6 μL of 10 mg.mL^−1^ Sinapic acid (Sigma) in 50% (*v*/*v*) acetonitrile and 5% (*v*/*v*) formic acid (LC/MS grade, Fisher, Hampton, NH, USA). The data was acquired in Linear Mid Mass Positive mode using a 5800 MALDI-TOF/TOF mass spectrometer (AB Sciex, Framingham, MA, USA) and TOF/TOF Series Explorer Software v.4.1.0 (AB Sciex). External calibration was performed using Protein MALDI-MS Calibration Kit (MSCAL3, ProteoMass, Merck).

### 2.4. Spectroscopic Techniques

#### 2.4.1. UV Visible Spectroscopy

UV-visible spectra of ImdcA and PdcA were recorded in a UV-1800 spectrophotometer (Shimadzu, Gamby, OR, USA) in the 250 to 750 nm wavelength range. While PdcA was prepared in 20 mM Tris-HCl buffer (pH 9.0) containing 100 mM KCl and 10 mM sodium cholate, ImdcA was prepared in 20 mM Tris-HCl buffer (pH 9.0) containing 100 mM NaCl and 0.2% SB12. UV-visible spectra were acquired for both the oxidized and the reduced state of the proteins. The reduction of the proteins was obtained by the addition of sodium dithionite. 

#### 2.4.2. EPR Spectroscopy

For the EPR experiments the ImdcA solution was prepared in 20 mM potassium phosphate buffer (pH 9) with 100 KCl and 0.5% Triton X-100, and PdcA prepared in 20 mM Tris-HCl buffer (pH 9) containing 50 mM KCl and 10 mM sodium cholate. EPR spectra were recorded on a Bruker ESP 380 spectrometer equipped with an ESR 900 continuous-flow helium cryostat (Oxford Instruments, Oxfordshire, UK) The conditions used in these experiments were: a temperature of 7 K and 18 K for ImdcA and PdcA, respectively; a microwave frequency of 9.39 GHz; an amplitude modulation of 1.0 mT; and a microwave power of 2 mW.

#### 2.4.3. NMR Spectroscopy

1H-1D-NMR spectra were collected at 25 °C on an Avance II+ 500 MHz NMR spectrometer (Bruker, Rheinstetten, Germany) equipped with a 5 mm TCI C/N Prodigy Cryo probe. The ImdcA sample was prepared in 20 mM Tris-HCl buffer (pH 9.0) containing 100 mM NaCl and 0.2% SB12 containing 10% ^2^H_2_O (99.9 atom%), while the PdcA sample was prepared in 20 mM Tris-HCl buffer (pH 9.0) with 100 mM KCl and 10 mM sodium cholate containing 10% ^2^H_2_O (99.9 atom%). For solvent suppression and enhancement of the paramagnetic signals, a SuperWEFT pulse sequence was applied. The NMR spectra were processed and analyzed using Bruker TopSpin 4.0 software.

### 2.5. Electrochemical Measurements of PdcA and ImdcA

Electrochemical measurements were carried out at 25 °C using a single chamber three-electrode system cell configuration consisting of a pyrolytic graphite edge (PGE) working electrode (IJ Cambria Scientific, Llanelli, UK), an Ag/AgCl (3 M KCl) reference electrode (IJ Cambria Scientific) and a graphite rod counter electrode (P46101-CMG, Morganite Luxembourg S.A., Luxembourg). Cyclic voltammetry (CV) experiments were acquired in a Autolab PGSTAT2014 potentiostat (Metrohom Autolab s.v., Utrecht, The Netherlands). Prior to use, the PGE electrode was polished with aqueous Al_2_O_3_ slurry (1.0 μm), rinsed with water, and dried with a tissue before being exposed to the protein. The proteins were deposited on the surface of the working electrode by drying 1 μL of each protein sample (50 µM) with constant gas nitrogen flow. The buffers used in these experiments were prepared by mixing 5 mM of HEPES (Sigma Aldrich), 5 mM of MES (Sigma Aldrich) and 5 mM of TAPS (Sigma Aldrich) and 100 mM KCl. The desired pH values were adjusted with 1 M NaOH or HCl solutions. The CV experiments were acquired three times in the scan range of −800 mV to +200 mV (vs. Ag/AgCl 3 M KCl). The baseline was obtained in similar conditions but without depositing the protein on the electrode. QSoas program (version 1.0) available at https://bip.cnrs.fr/groups/bip06/software/ [42] was used to subtract the capacitive current and suppress background noise of the raw electrochemical data. All potentials are reported with respect to a standard hydrogen electrode (SHE) by addition of 210 mV [43] to those measured.

### 2.6. Homology-Based Modelling

#### 2.6.1. CwcA

The structural model of CwcA was generated using the cryoelectron microscopy structure of cytochrome OmcS from *G. sulfurreducens* as a template (PDB code: 6EF8) [33]. This template was chosen because it has the highest sequence identity with the target among proteins with known structure (18% sequence identity) (Figure S1). The first 5 and the last 12 residues of CwcA were not modelled since they did not align with the template. A sequence alignment generated with the Robetta webserver (http://new.robetta.org/) was used as input to build the model, after manual curation to ensure that all the heme binding motifs were aligned. The model was built using the automodel class implemented in Modeller (version 9.22) [44] with the refinement degree set to slow. The final model corresponds to the one with the lowest value of the objective function, out of 20 generated structures. To analyze how CwcA monomers interact with each other, a model containing 3 adjacent protein molecules based on the template structure was built.

#### 2.6.2. OcwA

The structural model of OcwA from *T. ferriacetica* (Tfer_3193) was generated using the structure of OcwA from *T. potens* as a template (PDB code: 6I5B) [18]. Given that the two sequences have a very high sequence identity (99%), only the aminoacid residues that differ between the two proteins (three aminoacids from the holo-protein) were optimized, while the rest of the structure was considered to be identical to the template (Figure S2). The model was built using a mutation class implemented in Modeller (version 9.22) [44], which mutates and optimizes selected residues. The final model corresponds to the one with the lowest value of the objective function, out of 20 generated structures.

## 3. Results

### 3.1. Production of MHC Involved in EET of T. ferriacetica

The MHC ImdcA, PdcA and CwcA from *T. ferriacetica* were successfully cloned into expression vectors and heterologously over-expressed in the Gram-negative bacterium *E. coli*. While ImdcA and PdcA were further purified, CwcA could only be obtained in an insoluble state. The hexaheme cytochrome CwcA was extracted from the membrane fraction with urea under denaturating conditions, however its refolding was never achieved in solution. ImdcA was purified from the membrane fraction, while PdcA was isolated from the soluble fraction of *E. coli*. Recombinant proteins gave a single band in the SDS-PAGE gels between 35 and 48 kDa markers stained with Coomassie, demonstrating that both proteins were isolated from other cellular components (Figure S3). 

As expected for a protein associated with the inner-membrane, the N-terminal sequence for ImdcA (GIRD) showed that the signal peptide was not cleaved (Figure S4a). For PdcA, the N-terminal sequence (TAPEK) indicated that the protein was correctly processed in *E. coli*, with the signal peptide being cleaved in the position expected for processing in the native organism (Figure S4b). 

**Figure 1:**
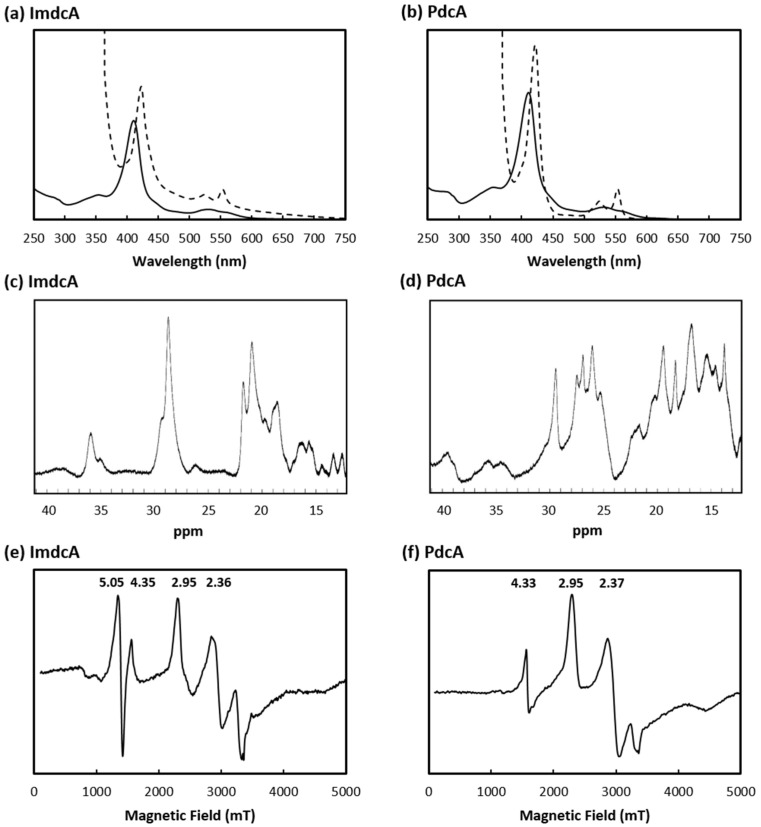
Spectroscopic characterization of ImdcA (left) and PdcA (right). (**a**,**b**) UV-visible spectra obtained in the oxidized (solid line) and reduced state (dotted line); (**c**,**d**) ^1^H-1D NMR spectra obtained at 25 °C in the oxidized state; (**e**,**f**) EPR spectra obtained at 9.39 GHz for the oxidized state. In both spectra, the signal at around *g* = 4.3 is expected to derive from iron (III) adventitiously present in the sample.

Mass spectrometry showed that the purified proteins have molecular masses of ~47.5 kDa and 42.6 kDa for ImdcA and PdcA, respectively, which agree with the calculated molecular mass of the apo-proteins (41.7 kDa and 36.5 kDa for ImdcA and PdcA, respectively) with the incorporation of the 10 hemes (~0.6 kDa per each heme). UV-visible spectra of both proteins showed the typical features of *c*-type cytochromes that contain hexacoordinated low-spin hemes (Figure 1a,b), with absorption peaks at 409 nm (Soret peak) and 530 nm in the oxidized state, and upon protein reduction, by the shift of the Soret peak to 421 nm, and the appearance of the β- and α-band at 525 and 552 nm, respectively. 

The ^1^H-1D-NMR spectra of both proteins in the oxidized state present signals outside the protein envelope, up to 40 ppm (Figure 1c,d), which indicates the presence of typical low-spin hemes axially coordinated by strong-field ligands such as histidines or methionines.

The EPR spectrum of ImdcA shows signals with at *g_max_* values of 2.95 and 2.36 (Figure 1e), while the spectrum of PdcA shows signals with *g_max_* values of 2.95 and 2.37 (Figure 1f). These are typical of hexacoordinated low-spin hemes with axial and rhombic electronic spin structure, that correlate with the presence of axial ligands of the iron that are parallel and perpendicular to each other, respectively [45]. ImdcA also contains a signal at *g* = 5.05 which can be tentatively assigned to a high-spin heme Fe(III) species.

### 3.2. Electrochemical Behavior of ImdcA and PdcA

**Figure 2:**
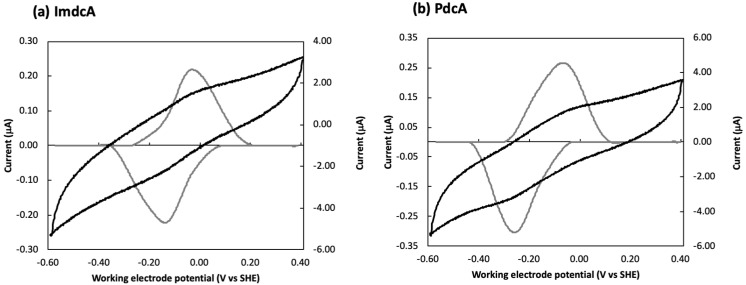
Cyclic voltammetry of (**a**) ImdcA and (**b**) PdcA: raw (black and right axis) and baseline-subtracted data (grey and left axis) of the voltammograms obtained at a scan rate of 200 mV/s at pH 6.

To study the redox properties of ImdcA and PdcA, purified proteins were adsorbed to the surface of a pyrolytic graphite “edge” electrode and CV were performed (Figure 2 and Figure S5). These data show that PdcA titrates at slightly lower reduction potentials when compared with ImdcA for the pH values tested, and that both proteins present redox-Bohr effect (i.e., the reduction potential of the redox centers changes with pH [46]) (Figure S5). This effect is usually observed in multicenter redox proteins, such as MHC [18,47,48], facilitating the full reduction of the protein, since the uptake of protons balances the electrostatic repulsion that arises from taking up multiple electrons. 

In a protein with multiple redox centers, as the case of ImdcA and PdcA, the voltammogram contains information about the redox behavior of each individual redox center [49]. The different shape observed for the voltammograms obtained at each pH indicate that the redox-Bohr effect does not impact the various hemes equally, as previously observed [50]. This is a consequence of the distance dependence of the electrostatic interaction between charged centers in MHC [51].

### 3.3. Structural Model of CwcA

**Figure 3:**
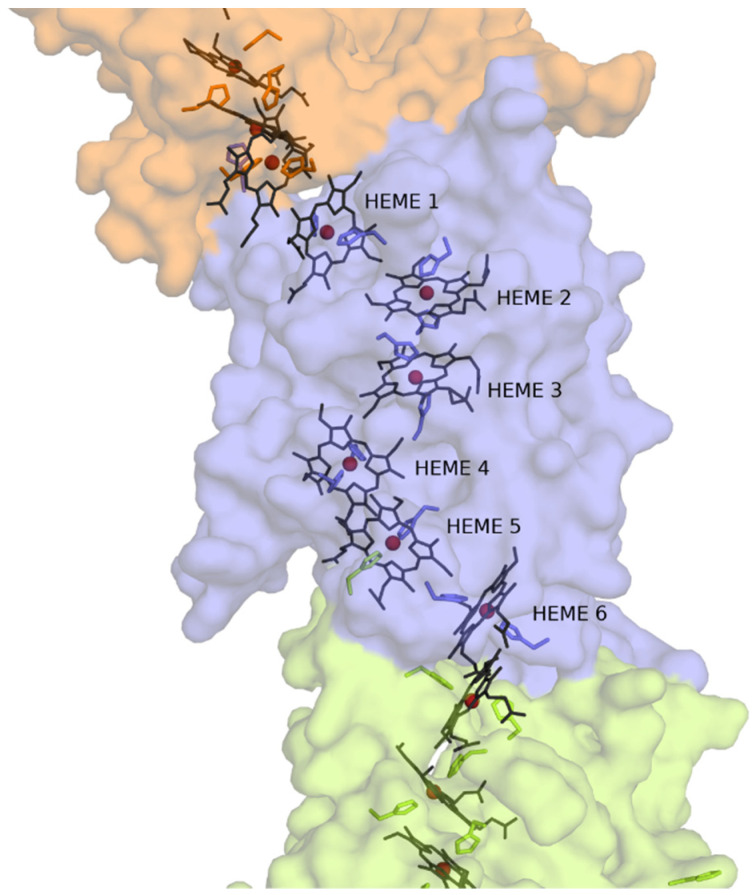
Homology-based model of the CwcA structure. Three CwcA molecules are displayed using molecular surface representations with different colors. The heme groups are represented as grey sticks with the Fe atoms displayed as dark red spheres and the coordinating histidines are represented as sticks of the same color as the respective molecular surface.

The structure of cytochrome OmcS from *G. sulfurreducens* was used as a template to build a structural model of CwcA. Although the sequences of the two proteins have a modest identity (18% of the residues are identical and 41% are either identical or have similar properties, Figure S1), the structures of two homologous MHC are usually considerably more conserved than the respective sequences [52,53]. The homology-based model of CwcA generated using the available structure of OmcS suggests that the protein can adopt a polymer-like structure similar to that observed in OmcS, where several protein molecules are stacked on top of each other forming a nanowire (Figure 3). Each CwcA molecule contains 6 hemes, coordinated by two histidines. This model indicates that one of the hemes (heme 5 in Figure 3) may be coordinated by a histidine belonging to a different cytochrome subunit, as occurs in OmcS of *G. sulfurreducens* [33]. The hemes in the protein model are packed within 3.5–6 Å of each other which is sufficiently close to guarantee fast intramolecular electron transfer along the polymer [47,54], a necessary requirement for efficient electron transfer within the respiratory pathway. Given the identity between the two sequences, the proposed model provides insights into the architecture and the mode of action of this protein, but does not to deliver a detailed prediction structure of CwcA. In the case of ImdcA and PdcA the homology with MHC of known structure was too low to support the calculation of structural models.

## 4. Discussion

In this work ImdcA and PdcA from *T. ferriacetica* were produced and biochemically characterized. The electrochemical characterization of ImdcA and PdcA shows that the window within which these proteins are electrochemically active spans more than 400 mV (Figure 2). Although the complexity of the systems, with ten hemes in each protein, precludes the determination of the reduction potential of each individual heme by these methods [7,49], it is clear from the electrochemical data that the distribution of the potentials of the various hemes differs, in particular with pH (Figure S5). Both proteins present spectroscopic properties typical of *c*-type cytochromes with hexacoordinated low-spin hemes, as observed for other MHC involved in EET pathways [18,27,47,48,53,55]. The aminoacid sequence of PdcA (Figure S4) suggests that only nine of the hemes are bis-histidinyl axial coordinated, and that probably one heme may contain a methionine as the distal coordinated ligand. In contrast, although ImdcA contains enough histidines for all the hemes to be hexacoordinated with two histidines, the EPR spectrum suggests that one of the hemes may be high-spin. Indeed, high-spin hemes in inner-membrane MHC, such as ImdcA, are often involved in the interaction with quinones from the quinone pool in the inner-membrane [56,57]. The range of redox potentials in which ImdcA and PdcA are electrochemically active is similar to that of MHCs of other electroactive organisms and adequate to extract electrons from low-potential quinone pools such as menaquinones [58,59]. This fits with the proposal that ImdcA oxidizes the quinone pool in the membrane and transfers the electrons to the soluble decaheme cytochrome PdcA present in the periplasmic space [34]. PdcA can then transfer the electrons outside of the cell, to OcwA or external electron acceptors, through CwcA that is embedded in the peptidoglycan layer (see below). The experimental determination of the electrochemical active range of CwcA could not be obtained but the homology model showing bis-histidine axial coordination of the hemes suggests that the reduction potentials will be in a similar range to those observed for PdcA and ImdcA [27,48]. The high sequence homology between OcwA from *T. potens* and *T. ferriacetica* (520/525 aminoacids) argues that their mode of action, redox behaviour, as well as their structural properties are very similar (Figure S2). Overall, ImdcA, PdcA CwcA and OcwA are redox active in a similar range of electrochemical potential (between +100 mV to −300 mV vs. SHE). As observed for other electroactive organisms [48], this range provides the necessary electrochemical window to establish connection between the cell metabolism and extracellular acceptors of higher potential such as iron minerals, maintaining a favorable driving force along the whole chain.

**Figure 4:**
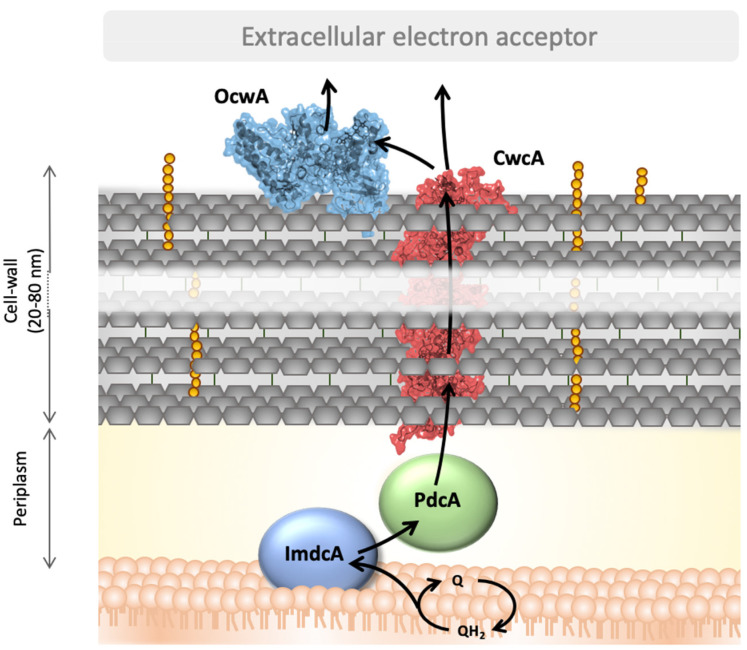
Representative scheme of the EET pathway of *T. ferriacetica* and the proteins involved. Cytochrome representations were made with PyMOL using the homology-based model of CwcA and OcwA. The correct orientation of both proteins is not known, and the scheme represents only one possibility of their position. MHC ImdcA and PdcA are represented as solid spheroids. The arrows indicate the electron flow from the quinone pool to the reduction of extracellular electron acceptors.

CwcA was not purified in this work due to the difficulties in stabilizing the protein in a soluble form. This experimental observation supports the computational homology model showing CwcA as capable of forming long polymers as experimentally observed for OmcS. Based on this structural model, we propose that CwcA can be arranged with similar structural features within the peptidoglycan wall of the Gram-positive bacterium where several protein molecules are stacked on top of each other forming a nanowire (Figure 3). This arrangement allows the hemes to be close to each other, guaranteeing fast intramolecular electron transfer within the proteins across the cell-wall, and probably beyond. Since the thickness of the peptidoglycan can vary between 20 and 80 nm [16,24], around 4 to 16 CwcA molecules can be arranged end to end to span the thickness of the cell-wall. This enables CwcA to emerge at the cell surface to transfer electrons to OcwA present at the cell surface, or directly to electron shuttles or insoluble electron acceptors. This fits with the current model of organization responsible for EET in Gram-positive bacteria belonging to the *Thermincola* genus [34,60] (Figure 4). A flavin-based transfer mechanism proposed for other Gram-positive bacteria [61] cannot be however excluded for this genus. Although this type of EET process has never been observed in *Thermincola* bacteria, genes involved in the flavinylation of proteins [62] are present in the genome of *T. potens*, suggesting that flavin mediated EET can also occur in *Thermincola*.

## 5. Conclusions

In contrast with Gram-negative bacteria, for which the understanding of the molecular bases for EET processes is more advanced [7,29], Gram-positive bacteria have a cell surface of very distinct nature composed by a thick peptidoglycan layer (20–80 nm). In this work, the production and characterization of the key components of the EET pathway of the Gram-positive bacterium *T. ferriacetica* allowed the preliminary characterization of their electron transfer processes. Indeed, the study of ImdcA and PdcA from *T. ferriacetica* showed that these proteins have the redox properties necessary to transfer electrons from the quinone pool in the membrane to the cell surface, for the reduction of electron acceptors including iron oxides, electron shuttles or electrodes. In fact, the overall reduction potential window by which these proteins becomes reduced are very similar to those of mesophilic organisms. This probably reflects the thermodynamic constrains given by the potentials of bioenergetic metabolic intermediates (e.g., NADH) and the potentials of the insoluble extracellular acceptors present in the natural environments colonized by these organisms. This study provides guidance for structural biology studies and detailed characterization of the electron transfer processes of these proteins. Only with this information will it be possible to fill in the details that are crucial to capitalize on the advantageous properties of thermophilic organisms to improve BES and increase the production of electricity or other valuable commodities from waste streams.

## Data Availability

Not applicable.

## Supplementary Materials

The following are available online at https://www.mdpi.com/2076-2607/9/2/293/s1, Table S1: Primers’ sequence used in this work, Figure S1: Sequence alignment used to build the CwcA homology based mode, Figure S2: OcwA from *T. ferriacetica*. Figure S3: SDS-PAGE of (a) ImdcA and (b) PdcA stained with blue-safe, Figure S4: Aminoacid sequence of (a) ImdcA and (b) PdcA, Figure S5: Raw voltammogram obtained by cyclic voltammetry for (a) ImdcA and (b) PdcA.
