# Supplementary material for: Crossing the Wall: Characterization of the Multiheme Cytochromes Involved in the Extracellular Electron Transfer Pathway of Thermincola ferriacetica
Source: Microorganisms. 2021 Jan 31;9(2):293. doi: 10.3390/microorganisms9020293 (PMC7911101; doi:10.3390/microorganisms9020293)
Supplement: Supplementary file 1 [file microorganisms-09-00293-s001.pdf]

Supplementary Materials

Table S1. Primers' sequence used in this work.

**Primers**	**Sequence (5' --> 3')**
Forw_Tfer0070	TAAGAAGGAGATATACATCCCATGGGTATCCGAGACAAATTTAAGTTT
Rev_Tfer0070	CTTGAACCAGTGACACTTGTTGCAATAGAGACCGG
strep_forw	TTCTACGCGTTGGAGTCATCCTCAGTTTGAGAAGTGAGTTTAAAC
strep_rev	GTTTAAACTCACTTCTCAAACTGAGGATGACTCCAACGCGTAGAA
Tfer1887_mut1_forw	TCCATGTCTTACCTGCCATAAAGGGTATGATCCCCAGC
Tfer1887_mut2_forw	CACGAGGCTGCCAAGGTACCTTGTCTATCTTGC
Tfer1887_mut3_forw	TGGCAATATCGCCGAGCGTAAGGTTACTACGAGACCTGA
Tfer1887_mut4_forw	GACTTGATTGCTGATTTTGCACGGGCTAACAGCTACTGC
Tfer1887_mut5_forw	CATGACGGTGCTTGGATGTCCAAACACCCGCAGATGGCTAAAGACAAGGGCCTGCAG
Tfer1887_mut6_forw	GCCCAGGCCGAATGTTACAGGTACTTATTGTAACACCTGCCACTGGTTCC
Tfer1887_mut1_rev	GCTGGGGATCATACCCTTTATGGCAGGTAAGACATGGA
Tfer1887_mut2_rev	GCAAGATAGACAAGGTACCTTGGCAGCCTCGTG
Tfer1887_mut3_rev	TCAGGTCTCGTAGTAACCTTACGCTCGGCGATATTGCCA
Tfer1887_mut4_rev	GCAGTAGCTGTTAGCCCGTGCAAAATCAGCAATCAAGTC
Tfer1887_mut5_rev	CTGCAGGCCCTTGTCTTTAGCCATCTGCGGGTGTTTGGACATCCAAGCACCGTCATG
Tfer1887_mut6_rev	GGAACCAGTGGCAGGTGTTACAATAAGTACCTGTAACATTCGGCCTGGGC
Forw_STC_0075	CCAACCGCATTTGCCGAAGAACCTTACTACC
Forw_STC_pBADthiof	TAAGAAGGAGATATACATCCCGTGAGCAAAAAACTATTAAG
Rev_Tfer0075	ATTAGCCTGCAACGTCGGCGTATTGTACT
Rev_STC_0075	GGTAGTAAGGTTCTTCGGCAAATGCGGTTGG

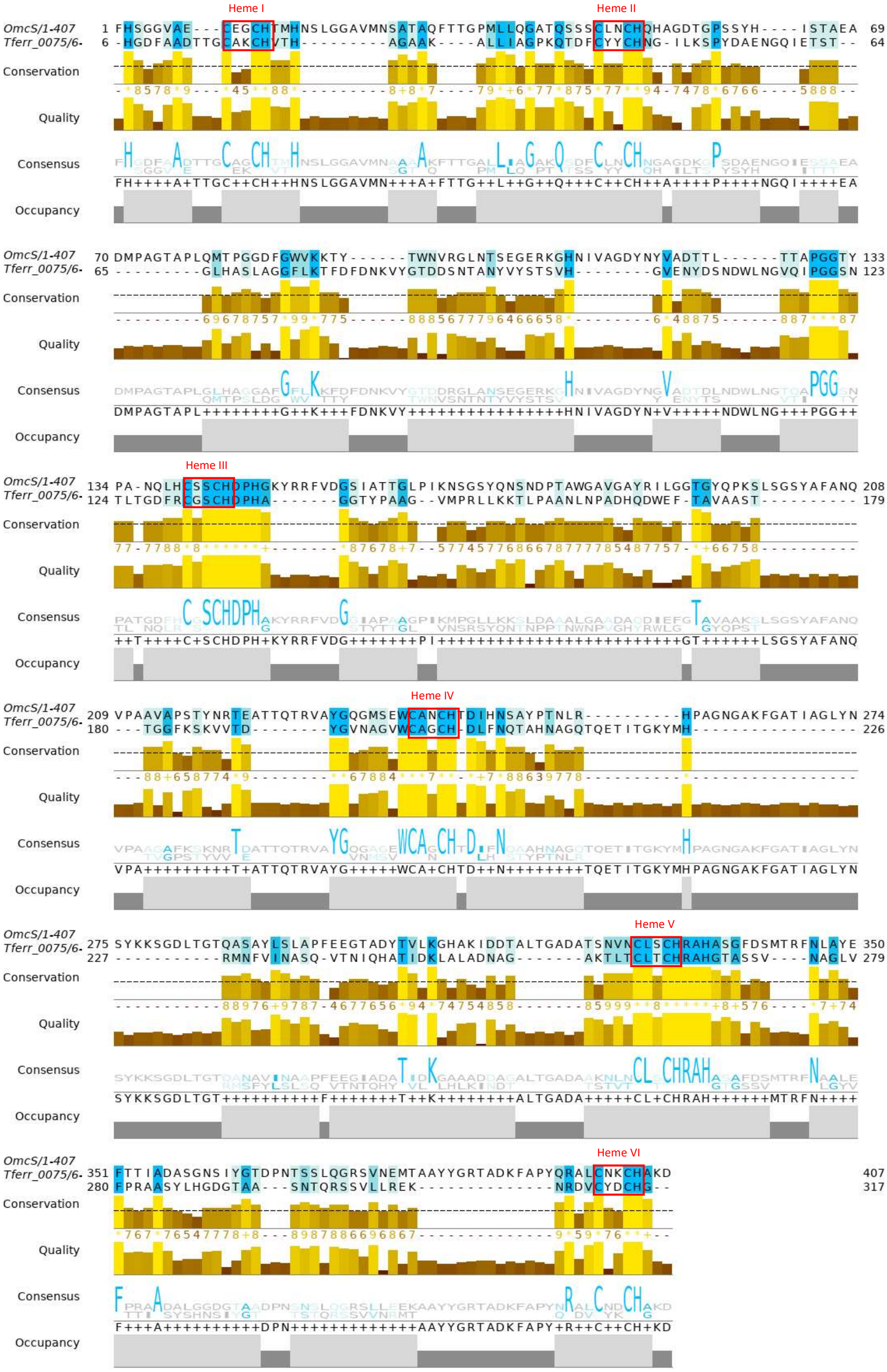

Figure S1. Sequence alignment used to build the CwcA homology based model. The figure was built with Jalview 2.11.0, highlighting the residues that are identical (turquoise) or have similar properties (light cyan). The sequence conservation, alignment quality, consensus residues and occupancy were annotated using Jalview annotation tool. The heme motifs are shown in red boxes.

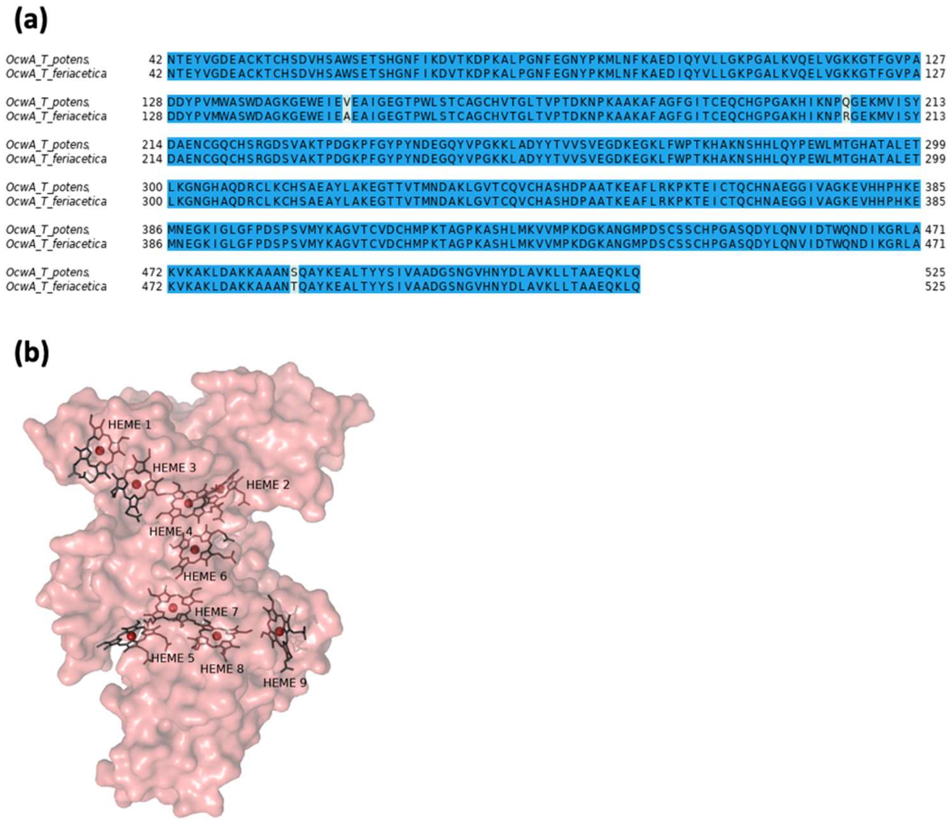

Figure S2. OcwA from *T. ferriacetica*. **(a)** Sequence alignment used to build the *T. ferriacetica* OcwA (Tfer_3193) homology based model. The figure was built with Jalview 2.11.0, highlighting the residues that are identical (turquoise). Only the residues that are present in the template structure are shown. **(b)** Homology-based model of the OcwA structure from *T. ferriacetica*. The structure of OcwA is displayed using a transparent molecular surface representation, coloured in pink. The heme groups are represented as sticks with the Fe atoms displayed as spheres.

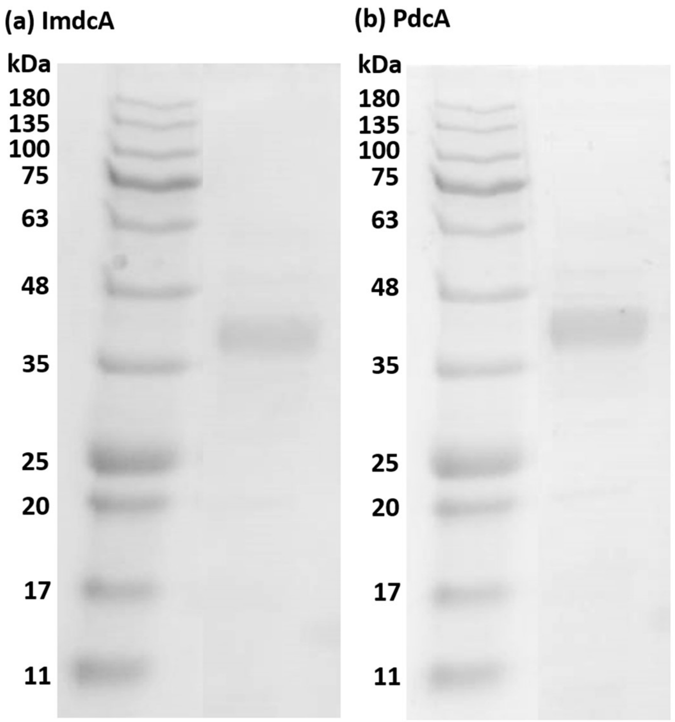

Figure S3: SDS-PAGE of (a) ImdcA and (b) PdcA stained with blue-safe.

(a) ImdcA

```
  1  MGIRDKFKFDLSKTEDKLKLFILVSGALLFILVAAVAGISLTMSPEFCVLCHDAMQPEYV   60
 61  TWKVSSHSNIRCVDCHMEPGVVNILIEKVMASKHLINYAITKEYKEEKPLHMKNELPSHL  120
121  CEKCHNVKNRNFTLSGDLIVPHELHGEKGVGCVKCHSGVAHGNIYKRGVSVGDIGAWTLE  180
181  DGKKNMAKQFTQPDMDVCVECHMNPAKFGVQGVKSVTFRCEACHKSIFTPENHKDKGW    238
239  TSQGLHGVSAESGDKEFKGCVMCHSIGVKTEKIATGNKVKDFAWGNQFCSSCHAKLPPSH  298
299  AQRDVWMPNHKKVVATKGMKNCEACHSLKAPEGKVSAPAGLYCNKCHWFK
```

(b) PdcA

```
  1  MLKRLHKFSLKKVMLLTALVLLALLVVGCSQQTSTAPATAPEKETSGDATQKQDTQAGGA   60
 61  TPTKVSDTEIAQTQCTECHEMWPEIATWQTSVHANVPCLTCHKGYDPQQNKSAHDSGSFQ  120
121  KPIAIRNNPVSDDACRSCHAMQNRLATLLPDLIAPPHEKHEAAKVPCLSCHRFVTHGNIAE  180
181  RKVTTRPEYSDYSKWSPQLAKQAAPQVMRRPNMFVCINCHEQRKVTTKCAACHYYPDRKS  240
241  LPSHENPEWKVIHGREGRKDVNNCAKCHYDKESQKFATPSTGDLIADFARANSYCYGCHL  300
301  KRPANHDGAWMSKHPQMAKDKGLQNCFACHDKNQPRPNVTGTYCNTCHWFQDPKPAA     357
358  EQAQKK
```

Figure S4. Aminoacid sequence of (a) ImdcA and (b) PdcA. The signal peptide of PdcA predicted to be cleaved (predicted using SignalP-5.0 Server at http://www.cbs.dtu.dk/services/SignalP/) are represented in red. The heme binding motifs are highlighted in yellow.

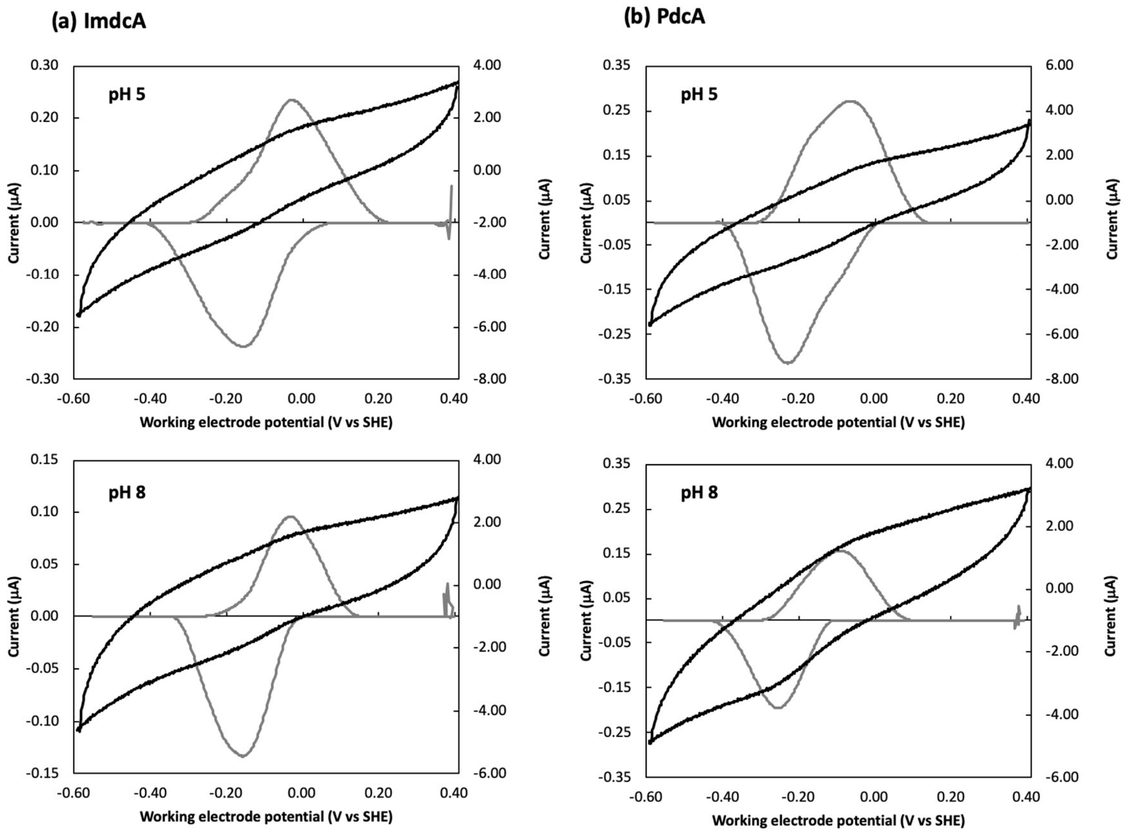

Figure S5. Raw (black and right axis) and baseline-subtracted data (grey and left axis) voltammogram obtained by cyclic voltammetry for (a) ImdcA and (b) PdcA at a scan rate of 200 mV/s at pH 5 and 8.
